# Simplified Iohexol-Based Method for Measurement of Glomerular Filtration Rate in Goats and Pigs

**DOI:** 10.3390/biology10060461

**Published:** 2021-05-23

**Authors:** Maaike K. van Gelder, Jasper Stevens, Tobias T. Pieters, Koen R. D. Vaessen, Jaap A. Joles, Marianne C. Verhaar, Karin G. F. Gerritsen

**Affiliations:** 1 Department of Nephrology and Hypertension, University Medical Center Utrecht, Heidelberglaan 100, 3584 CX Utrecht, The Netherlands; m.k.vangelder-7@umcutrecht.nl (M.K.v.G.); T.T.Pieters-4@umcutrecht.nl (T.T.P.); J.A.Joles@umcutrecht.nl (J.A.J.); M.C.Verhaar@umcutrecht.nl (M.C.V.); 2 Department of Clinical Pharmacy and Pharmacology, University Medical Center Groningen, University of Groningen, Hanzeplein 1, 9713 GZ Groningen, The Netherlands; j.stevens@umcg.nl; 3 Central Laboratory Animal Research Facility, Utrecht University, Heidelberglaan 8, 3584 CS Utrecht, The Netherlands; K.R.D.Vaessen@uu.nl

**Keywords:** glomerular filtration rate, iohexol, plasma clearance, uremic animal model, goat, chronic kidney disease

## Abstract

**Simple Summary:**

To improve the treatment of patients with kidney disease, new therapies are being developed. Before being used on humans, such therapies need to be tested on animals with kidney disease because reduced kidney function may influence the safety and efficacy of the treatment. Using large animals for this purpose is important because they tolerate frequent blood sampling, which allows for repeated monitoring. Goats seem particularly suitable for the evaluation of novel hemodialysis therapies since they are docile, have easily accessible neck veins to obtain blood access and body weights comparable with humans. Currently, no simple method is available to measure kidney function in goats (with or without impaired kidney function). Therefore, we developed a simple method to measure the kidney function in goats and pigs, which is based on a single injection of iohexol and requires three blood samples. Subsequently, kidney function can be calculated using a formula derived from pharmacokinetic modelling. The measurement of kidney function using our simplified method is relatively easy to perform, reduces total blood sampling and eliminates the need for an indwelling bladder catheter as compared to existing methods that require continuous infusion of a substance and timed urine collection.

**Abstract:**

The preclinical evaluation of novel therapies for chronic kidney disease requires a simple method for the assessment of kidney function in a uremic large animal model. An intravenous bolus of iohexol was administered to goats (13 measurements in *n* = 3 goats) and pigs (23 measurements in *n* = 5 pigs) before and after induction of kidney failure, followed by frequent blood sampling up to 1440 min. Plasma clearance (CL) was estimated by a nonlinear mixed-effects model (CL_NLME_) and by a one-compartmental pharmacokinetic disposition model using iohexol plasma concentrations during the terminal elimination phase (CL_1CMT_). A simple method (CL_SM_) for the calculation of plasma clearance was developed based on the most appropriate relationship between CL_NLME_ and CL_1CMT_. CL_SM_ and CL_NLME_ showed good agreement (CL_NLME_/CL_SM_ ratio: 1.00 ± 0.07; bias: 0.03 ± 1.64 mL/min; precision CL_SM_ and CL_NLME_: 80.9% and 80.7%, respectively; the percentage of CL_SM_ estimates falling within ±30% (P30) or ±10% (P10) of CL_NLME_: 53% and 12%, respectively). For mGFR_NLME_ vs. mGFR_SM_, bias was −0.25 ± 2.24 and precision was 49.2% and 53.6%, respectively, P30 and P10 for mGFR based on CL_SM_ were 71% and 24%, respectively. A simple method for measurement of GFR in healthy and uremic goats and pigs was successfully developed, which eliminates the need for continuous infusion of an exogenous marker, urine collection and frequent blood sampling.

## 1. Introduction

Chronic kidney disease (CKD) is an important health care problem affecting approximately 10–16% of the population worldwide [1,2]. To improve the outcome of patients with CKD, there is a need for novel therapies. The preclinical evaluation of such therapies requires a simple method for the assessment of kidney function in a uremic large animal model. Currently, no simple method is available to measure glomerular filtration rate (GFR) in a uremic large animal with a body weight similar to humans. Glomerular filtration rate (GFR) is considered as the best indicator of (residual) kidney function and it is an important biomarker in clinical and pre-clinical drug development, as kidney function is a determinant of the pharmacokinetics of many drugs. The use of a large animal model for this purpose is important because such animals tolerate frequent blood sampling, which allows repetitive measurements and thus assessment of plasma clearance (CL) as well as adequate monitoring of efficacy and safety. Goats seem particularly suitable for the evaluation of novel hemodialysis therapies since these animals are docile, have easily accessible neck veins and have body weights (70–90 kg) and distribution volumes comparable with humans [3]. In addition, pigs were used for the preclinical evaluation of novel therapies for CKD as these animals have similarities to humans in terms of bodyweight (and distribution volume) and renal anatomy and physiology [4,5,6,7].

Regulatory agencies, such as the European Medicines Agency (EMA), recommend that during drug development actual GFR is measured (mGFR) at least once [8,9]. GFR can be measured after infusion of an exogenous substance that is freely filtered by glomeruli, and that is not secreted, reabsorbed, synthesized or metabolized by the kidney. The gold standard for mGFR is inulin clearance [10], which requires a continuous infusion of inulin for several hours, a careful assessment of urinary inulin excretion and repeated blood sampling to monitor inulin steady-state plasma concentrations. This method is cumbersome, time-consuming and expensive. Moreover, the production of inulin has recently been discontinued.

Alternative markers for measurement of GFR are available that can be administered as a single bolus injection, including ^51^Cr-EDTA, ^99m^Tc-DTPA, iothalamate and iohexol [10]. The mGFR derived from renal excretion of ^51^Cr-EDTA and ^99m^Tc-DTPA are reliable methods to determine mGFR, but the use and storage of radioactive agents are restricted to nuclear facilities, which is relatively costly and impractical. Iohexol, a non-isotopic contrast agent, is currently the most widely used alternative marker for GFR measurement in humans due to its ease of use and favorable safety profile and physicochemical properties (molecular weight: 821 Da, protein binding: 1.5%, no renal secretion, metabolism or -reabsorption, negligible extra-renal clearance) [11]. Iohexol plasma clearance is calculated by dividing the administered iohexol dose by the area under the plasma iohexol disappearance curve (AUC) using non-compartmental or compartmental models and therefore accurately predicts GFR [10]. Following intravenous administration of iohexol, the plasma concentration–time profile of iohexol is characterized by a three-exponential curve; a rapid distribution phase, followed by a second slow distribution phase and a constant elimination phase. To accurately measure the plasma clearance of iohexol, frequent blood sampling is required to fully capture the iohexol plasma disappearance curve. To reduce the need for frequent blood sampling after bolus injection, many mathematical corrections were reported to calculate iohexol clearance based on linear regression using only two or three iohexol plasma concentration measurements in the elimination phase, e.g., from 180 min after bolus iohexol administration onwards [11]. In humans, the method developed by Bröchner–Mortensen is most widely applied [11,12], where the clearance is calculated based on the linear regression during the constant elimination phase after which a formula is applied to correct for the lacking distribution phases. By this approach, estimating iohexol plasma clearance after a single bolus injection of iohexol and limited blood sampling is relatively easy to perform, less time consuming, reduces total blood sampling volume and eliminates the need for an indwelling urinary catheter as compared to renal clearance methods that require continuous infusion of an exogenous marker and urine collection.

However, no simple method, such as the Bröchner–Mortensen formula, is available for the measurement of GFR in goats and pigs. Therefore, the primary aim of this study was to develop a simple method to measure GFR repeatedly by plasma clearance in goats and pigs with normal and impaired kidney function after a single bolus injection of iohexol.

## 2. Materials and Methods

### 2.1. Animals

White adult goats (Capra aegagrus hircus) (*n* = 3) weighing 48–84 kg were obtained from V.O.F. de Römer (Heythuysen, The Netherlands). Goats were screened for caprine arthritis encephalitis, caseous Lymphadenitis, paratuberculosis, and bovine virus diarrhoea, and vaccinated against Q-fever. Clinically healthy female pigs (Topigs Norsvin; *n* = 5) weighing 34–80 kg were obtained from Van Beek SPF varkens B.V. (Lelystad, the Netherlands). Pigs were screened for several common viral diseases (pseudorabies, porcine reproductive and respiratory syndrome, classical swine fever, African swine fever, transmissible gastroenteritis, influenza, porcine epidemic diarrhoea, porcine delta coronovirus, rotavirus, swine vesicular disease), bacterial infections (Mycoplasma hyopneumoniae, Actinobacillus pleuropneumonia, brachyspira hyodysenteriae, leptospira, Pasteurella multocida, brucella, MRSA, salmonella) and ectoparasites. After placement of a jugular line, animals were housed indoors in individual cages to prevent dislodgement of the line by other animals. Otherwise, animals were housed in groups. The temperature of the animal room was maintained between 18 and 21 °C and artificial lighting was provided. Goats were offered 300 g of dry feed (Kasper Faunafood, Woerden, The Netherlands) per day and pigs 1500 g of dry feed (9050 Maintenance diet for minipig, rich in crude fibre, Altromin International, Lage, Germany). Hay for goats, straw as bedding for both pigs and goats and water were provided ad libitum.

### 2.2. Experimental Design

GFR was measured in *n* = 3 goats (age, range: 6–24 months) and *n* = 5 pigs (3–8 months) before and after the induction of CKD within the same animal (2 measurements in *n* = 2 goats and 10 measurements in *n* = 4 pigs before induction of CKD, and 11 measurements in *n* = 3 goats and 13 measurements in *n* = 5 pigs afterwards). Uremia was established by embolization of (branches) of the renal artery using polyvinyl alcohol particles, an accepted method [13] aiming for embolization of ~80% of one kidney and complete embolization of the contralateral kidney. GFR measurements after embolization were performed once plasma urea and creatinine values had stabilized. Repeated experiments in the same animal before and after the induction of kidney failure were performed within a six-week time period to reduce intra-subject variation in GFR. In one goat, a three-month interval was present between the first and last GFR measurement during which clinical condition and plasma urea and creatinine concentrations were stable.

At least one day prior to GFR measurement, an indwelling venous catheter (silicone catheter 7 Fr, 60 cm, 2 moveable beads with injection cap, Access technologies, Chicago, IL, USA) was placed in the internal jugular vein under general anesthesia (premedication: midazolam 0.7 mg/kg i.m., ketamine 13 mg/kg i.m. and atropine sulphate 0.05 mg/kg i.m.; induction and maintenance: propofol 3 mg/kg i.v. and 3.5 mg/kg/h, respectively, and remifentanil 30 µg/kg/h i.v.) for iohexol administration and repeated blood withdrawal. Animals were awake during experiments. A single intravenous bolus of 1500 mg iohexol (5 mL, Omnipaque 300 mg/mL, GE Healthcare, Machelen, Belgium) was administered followed by flushing with sodium chloride 0.9%. A 3.5 mL venous blood sample was collected in a K2 EDTA tube with a gel separator (Vacuette^®^, Greiner Bio-One, Alphen aan den Rijn, The Netherlands) before bolus administration and after 5, 10, 20, 30, 60, 120, 180, 240, 300, 360, 480 and 1440 min for measurement of iohexol plasma concentrations. Blood samples were centrifuged within 2 h and plasma was aliquoted and stored at −80 °C until analysis.

### 2.3. Laboratory Analyses

Iohexol plasma concentrations were measured at the University Medical Center Groningen (Groningen, The Netherlands) using liquid chromatography-tandem mass spectrometry (Thermo Scientific Vanquish^TM^ UPLC system, Thermo Fisher Scientific, Waltham, MA, USA; Thermo Scientific^TM^ Quantiva^TM^ tandem quadrupole mass spectrometer, Thermo Fisher Scientific, San Jose, CA, USA) validated for goat and pig EDTA plasma as described previously [14]. In short, 50-µL of internal standard dissolved in trichloroacetic acid for deproteinization (10 mg of ^2^H_5_-iohexol in 50 mL trichloroacetic acid 10%) was added to 100-µL of plasma, calibration curve samples and quality controls. The samples were vortexed for 1 min prior to centrifuging at 9500× *g* for 5 min. After precipitation, 10 μL of supernatant was diluted 1:100 with water in an autosampler vial. After vortexing for 1 min, 40 μL was injected into the LC-MS/MS.

### 2.4. Statistical Analysis

To develop the simplified method to calculate iohexol plasma clearance, a correction factor was applied to a one-compartmental model, using iohexol plasma concentrations measured at 180, 240 and 300 min (i.e., the terminal elimination phase). The correction factor was derived by comparison of iohexol plasma clearance calculated by a one-compartment model with the “gold standard”, i.e., a nonlinear mixed effects model that was based on all available iohexol plasma concentrations.

#### 2.4.1. Nonlinear Mixed Effects Model to Determine Iohexol Clearance

As a reference method, the gold standard in population pharmacokinetic analyses was applied; a nonlinear mixed effects model (NLME), which was based on all available iohexol plasma concentrations (i.e., before bolus administration and 5, 10, 20, 30, 60, 120, 180, 240, 300, 480 and 1440 min afterwards). A stepwise approach was used to develop a pharmacokinetic model that accurately describes the observed iohexol plasma concentration profiles. Different structural models were explored, including one-, two- and three-compartment models with linear elimination processes. Parameter estimates, e.g., absolute iohexol plasma clearance (CL_NLME_), volume of distribution (V) and intercompartmental clearance (Q) were estimated using first-order conditional estimation with interaction. Impaired kidney function was assumed as a discrete covariate prior to model development (parameterized as multiplication by CL_NLME_). Variability in the PK parameters within individuals within one study occasion (i.e., interindividual variability, IIV) and variability in the PK parameters within individuals between study occasions (i.e., interoccasion variability, IOV) were incorporated in the model, assuming a log–normal distribution of the random effects on the model parameters [15]. Additive, proportional and combined residual variability models were tested. Bodyweight and species were explored as covariates that may explain IIV using correlation matrices of the empirical Bayes estimates of the parameters versus potential covariates. Significant covariates (*p* < 0.05) were taken forward in the model development. For the continuous covariate bodyweight, allometric scaling with and without fixed power coefficients was explored. For the discrete covariate species, separate population parameters were estimated (parameterized as multiplication by population parameter). Model selection and evaluation was based on the minimum objective function value (MOFV, using *p* < 0.05, e.g., >3.84 points decrease in MOFV), standard goodness-of-fit plots, residual standard error (RSE) of the population parameter estimates and the coefficient of variation (CV) of the IIV [16]. From this pharmacokinetic model, individual values of iohexol clearance (CL_NLME_) were used for further analysis.

#### 2.4.2. Simplified Method to Determine Iohexol Clearance

**Figure 1:**
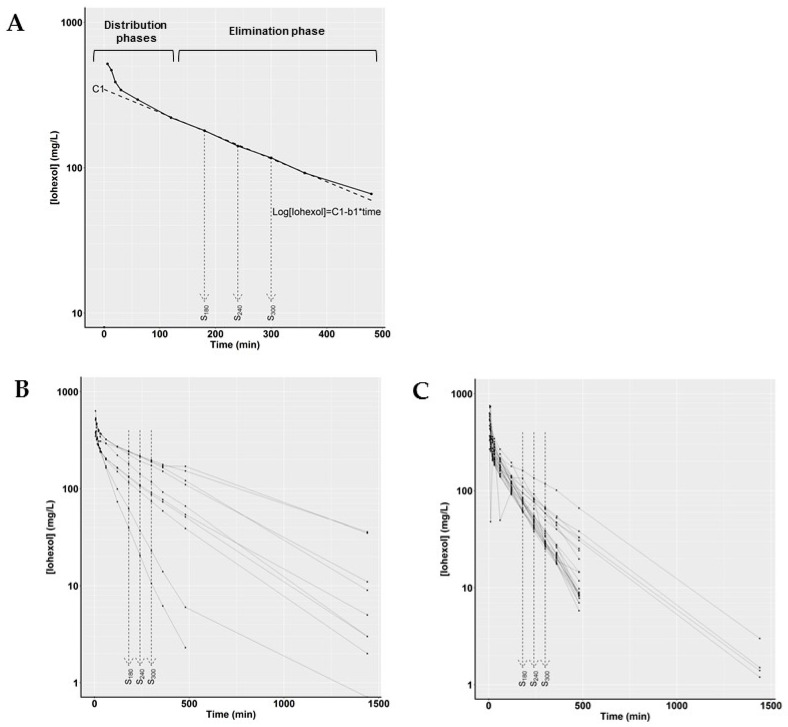
(**A**) Example of a typical plasma concentration–time profile after bolus administration of iohexol in a uremic goat (*n* = 1). Solid circles represent the iohexol plasma concentrations, the dotted line represents the linear regression (intercept; C_1_, slope; b_1_) over the log-transformed iohexol plasma concentrations taken at sample times 180, 240 and 300 min (S_180_, S_240_ and S_300_, respectively). (**B**,**C**) Plasma concentration–time profiles of all iohexol plasma clearance measurements after bolus administration of iohexol in goats (**B**) and pigs (**C**). Circles represent the iohexol plasma concentrations, the dotted lines represent the iohexol plasma concentrations taken at S_180_, S_240_ and S_300_, which were used for linear regression analysis according to the simplified method.

Following intravenous administration of iohexol, the plasma concentration–time profile of iohexol is characterized by a three-exponential curve; a rapid distribution phase, followed by a second slow distribution phase and a constant elimination phase (Figure 1). The intercept (C_1_) and slope of the constant elimination phase (b_1_) were calculated by linear regression in R (lm(log[concentration]~time)) using iohexol plasma concentrations measured at 180, 240 and 300 min (terminal elimination phase, Figure 1a).

The amount of iohexol infused (Q_inf_), the intercept and slope and the terminal elimination phase were used to calculate the clearance assuming a one-compartmental pharmacokinetic disposition model (CL_1CMT_, Equation (1) [12]).

(1) CL1CMT=QinfC1b1

The relationship between individual values of CL_NLME_ and CL_1CMT_ was explored by linear, non-linear or two-segmented linear relationships where the breakpoint was estimated by the software. The most appropriate relationship was chosen based on goodness-of-fit, numerical diagnostics and Blant–Altman plots. Bias was obtained from the Bland–Altman analysis by estimating the mean difference and the standard deviation of the differences between CL_NLME_ and CL_1CMT,_ and precision (2 × standard deviation/mean × 100%) was calculated for both methods. Finally, we considered the accuracy within 30% and 10%, i.e., the percentage of CL_SM_ estimates falling within ±30% (P30) or ±10% (P10) of CL_NLME_. The resulting equation provides a simplified method (SM) to calculate the individual CL as a function of CL_1CMT_ (CL_SM_).

To calculate mGFR, iohexol plasma clearance was normalized to a standardized body surface area (BSA) of an animal of 70 kg (Equation (3) for goats, Equation (5) for pigs). The BSA of goats was calculated using previously published methods by Saito (Equation (2)) [17]. For pigs, the BSA was calculated, as described by Kelly (Equation (4)) [18].

BSA_SAITO_ = WGT_i_^0.62^ × 1147.7 / 10000 (2)

GFR_SAITO_ = CL_n_ / BSA_SAITO_ × 1.43 × 1000 (3)

where 1.43 is the BSA in m^2^ for a typical 70 kg goat.

BSA_Kelly_ = WGT_i_^0.656^ × 734 / 10000 (4)

GFR_Kelly_ = CL_n_ / BSA_Kelly_ × 1.19 × 1000 (5)

where 1.19 is the BSA in m^2^ for a typical 70 kg pig.

For each animal, mGFR was calculated according to the NLME method and the SM, based on their respective clearances. Bias was obtained from a Bland–Altman analysis and precision was calculated for both methods as were the P30 and P10 for mGFR_SM_.

### 2.5. Software

NLME was performed in NONMEM 7.3 (Icon, Dublin, Ireland) [19]. All data processing, statistical analyses and graphical representations were performed in R version 3.5.3 (The R Foundation for Statistical Computing, Vienna, Austria) [20], using the tidyverse, segmented, blandr and ggplot2 packages.

## 3. Results

### 3.1. Statistical Analysis

#### 3.1.1. Data

**Figure 2:**
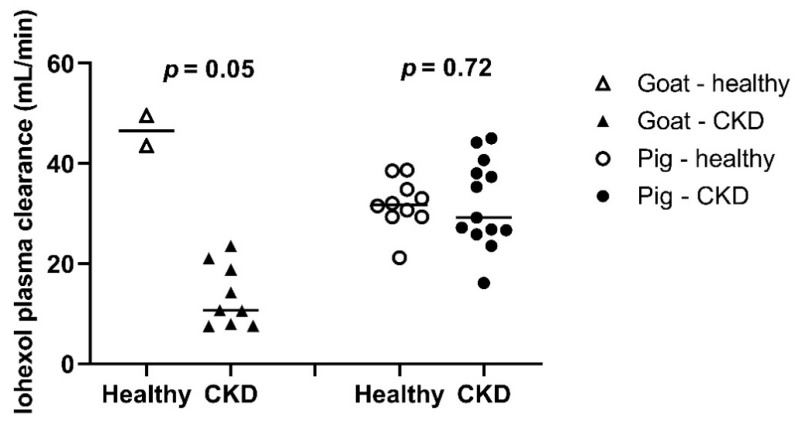
Iohexol plasma clearances after bolus administration of iohexol in goats and pigs (3–8 months of age) before and after induction of CKD within the same animal (2 measurements in *n* = 2 goats and 10 measurements in *n* = 4 pigs before induction of CKD, and 11 measurements in *n* = 3 goats and 13 measurements in *n* = 5 pigs afterwards). *p* values were calculated using a Student’s paired t-test.

In total, 13 iohexol clearance measurements were performed in *n* = 3 goats (2 measurements in healthy goats, 11 measurements in uremic goats) and 23 measurements were performed in *n* = 4 pigs (10 measurements in healthy pigs, 13 measurements in uremic pigs). Iohexol plasma concentrations below the lower limit of quantification were excluded for statistical analysis (14 (3%) of 469 samples in total). One extreme outlier was excluded (iohexol concentration was 11,317 mg/L). During two clearance measurements, maximum iohexol plasma concentration was reached after 20 min and 180 min, respectively. As this concentration–time profile is illogical after intravenous bolus infusion and suggests extravasation of iohexol, these clearance measurements were excluded from further analysis. Iohexol plasma clearances before and after induction of CKD are presented in Figure 2.

#### 3.1.2. Nonlinear Mixed Effects Model to Determine Iohexol Clearance

In the development of the structural pharmacokinetic model, a three-compartment model (MVOF = 2811.5) proved significantly better at describing the data compared to a one- (MVOF = 3139.0) or two-compartment (MVOF = 2896.6) model. Besides the a priori parameter for impaired kidney function, IOV was identified on CL and IIV on the peripheral volume of distribution. A proportional error model proved best fit for purpose. The incorporation of bodyweight as allometric scaling with fixed power coefficients improved the goodness-of-fit plots. In addition, the scaling of CL between the species improved the model significantly. Bodyweight and species were therefore included in the model as covariates.

In general, the population and individual trend of the data are well captured by the model, both for goats and pigs, as both the population and individual data lie randomly scattered around the line of unity (Figure S1). The goodness-of-fit plots show that the conditional weighted residuals with interaction seem to increase over time, although still within acceptable levels. The population parameter estimates (Table S1) were estimated with high precision as indicated by their low RSE, ranging from 4.6 to 13.5%. Most importantly, the individual iohexol plasma concentration–time profiles were accurately described by the model.

#### 3.1.3. Simplified Method to Determine Iohexol Clearance

**Figure 3:**
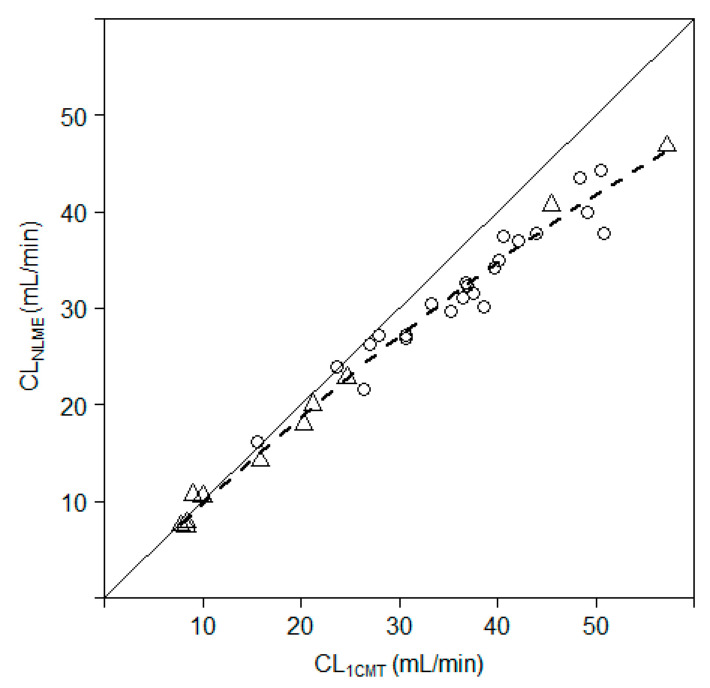
Comparison iohexol plasma clearance based on NLME (CL_NLME_) and a one-compartment pool model (CL_1CMT_). Dotted line, nonlinear regression line; solid line, line of unity; triangles, goat; circles, pig.

The relationship between individual values of the CL_NLME_ (range; 7.35–46.83 mL/min) and CL_1CMT_ (range; 7.82–57.28 mL/min) was explored by linear, non-linear or segmented linear relationships. The linear regression was statistically significant (*p* < 0.01) with a residual standard error (RSE) of 1.75 on 32 degrees of freedom (DF) and an adjusted R^2^ of 0.976 and Akaike information criterion (AIC) of 138.49. The standardized residuals vs. fitted values ranged from approximately −6 to 3, although most values lie above 0. Forcing the linear model through the origin worsened the fit (*p* < 0.01) with a RSE of 2.03 (DF = 33) and AIC of 147.49, despite an improvement of the adjusted R^2^ to 0.995. The bias in the goodness-of-fit remained. The non-linear model (CL = ax − bx^2^) improved the fit (*p* < 0.01, Figure 1) to an RSE of 1.67 (DF = 32) and AIC of 135.23. The standardized residuals vs. fitted values ranged from approximately −2.5 to 2 and most values were now randomly scattered around 0. The segmented model (*p* < 0.01), with an estimated breakpoint of 23.7 mL/min, did not outperform the nonlinear model, with an RSE of 1.71 (DF = 30), adjusted R^2^ of 0.98 but a higher AIC of 138.82. There was no visible improvement in the standardized residuals vs. fitted values. The nonlinear model was considered to be most fit for purpose, as there was no apparent improvement using the segmented model, which is also more difficult to interpret and apply. The relationship between CL_1CMT_ and CL_NLME_ is depicted in Figure 3.

The formula for the calculation of the plasma clearance according to the simplified method (CL_SM_) is depicted in Equation (6). The constants 1.006348 and 0.003437 are statistically different from 0 (*p* < 0.0001) with standard errors of 3.6 × 10^−2^ and 8.4 × 10^−4^. In short, one can now use three blood samples (t = 180, t = 240 and t = 300 min) and equation 1 to estimate CL1_CMT_, and calculate CL_SM_ according to Equation (6).

(6) $$CL_{\mathrm{SM}}=1.006348\times CL_{1\mathrm{CMT}}-0.003437\times CL_{1\mathrm{CMT}}^{2}$$

#### 3.1.4. Evaluation of the Simplified Method versus Reference Method

**Figure 4:**
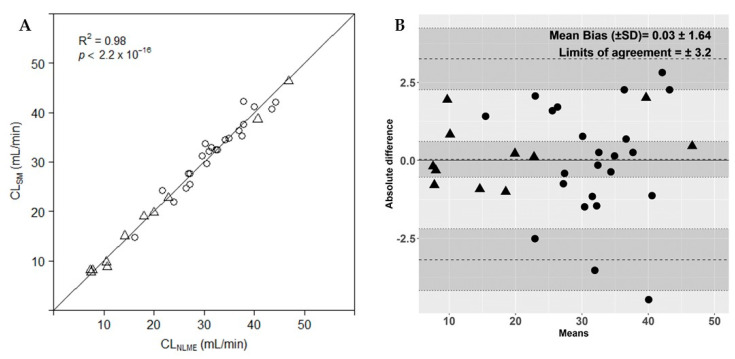
(**A**) Comparison of the measured iohexol plasma clearance based on a nonlinear mixed effects model (CL_NLME_) and calculated iohexol plasma clearance based on the simplified method (CL_SM_) in goats (triangles) and pigs (circles). Solid line, line of unity. (**B**) Bland–Altman plot of agreement between iohexol plasma clearance determined by a nonlinear mixed effects model and by the simplified method for goats (triangles) and pigs (circles). Dotted lines represent bias, upper and lower limits of agreement. Grey shaded areas represent the 95% confidence intervals of the bias, upper and lower limits of agreement.

Figure 4a shows the values of the measured iohexol plasma clearance based on a nonlinear mixed effects model (CL_NLME_) versus the calculated iohexol plasma clearance based on the simplified method (CL_SM_, range; 7.67–46.37 mL/min). All values follow the line of unity with a mean (±SD) CL_NLME_/CL_SM_ ratio of 1.00 ± 0.07. Similarly, Bland–Altman analysis (Figure 4b) showed that the mean bias (±SD) is 0.03 ± 1.64 with upper and lower limits of agreement of ±3.2, resulting in a precision of agreement between the two methods of 11.6%. The precision of the CL_NLME_ and CL_SM_ were 80.9% and 80.7%, respectively (Table 1). The P30 and P10 of CL_SM_ were 53% and 12%, respectively. The data are randomly scattered around 0, indicating no consistent bias of one method versus the other. There is no evidence of a species-dependent bias.

#### 3.1.5. mGFR Results

**Figure 5:**
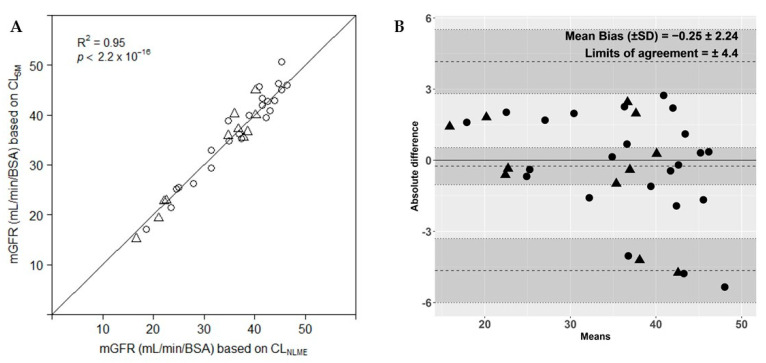
(**A**): Comparison of measured glomerular filtration rate (mGFR) based on clearance determined by a nonlinear mixed effects model (CL_NLME_) vs. the mGFR based on clearance determined by the simplified method (CL_SM_). All GFR values were adjusted for body surface area (BSA), assuming a BSA of 1.43 m^2^ in goats and 1.19 m^2^ in pigs. Solid line, line of unity; triangles, goat; circles, pig. (**B**): Bland–Altman plot of agreement between measured glomerular filtration rate (mGFR) based on plasma clearance determined by a nonlinear mixed effects model (CL_NLME_) and mGFR based on clearance determined by the simplified method (CL_SM_) for goats (triangles) and pigs (circles). Dotted lines represent bias, upper and lower limits of agreement. Grey shaded areas represent the 95% confidence intervals of the bias, upper and lower limits of agreement.

Figure 5a shows the values of the mGFR based on CL_NLME_ (range; 16.6–46.3 mL/min/BSA) versus the mGFR based on CL_SM_ (range; 15.2–50.7 mL/min/BSA), assuming a BSA of 1.43 m^2^ in goats and 1.19 m^2^ in pigs. All values follow the line of unity with a ratio of 1.00 ± 0.06. The Bland–Altman analysis (Figure 5b) showed that there is a bias of −0.25 ± 2.24 with upper and lower limits of agreement of ±4.4, resulting in a percentage of error between the two methods of 12.5%. The precisions of mGFR based on CL_NLME_ and mGFR based on CL_SM_ separately were 49.2% and 53.6%. The P30 and P10 of CL_SM_ were 71% and 24%. Again, there is no specific bias towards species.

There are no data in the 95 confidence interval of the upper limit of agreement, indicating a slight bias of one approach versus the other, where the simplified method seems to slightly overestimate the mGFR in 2 goats and 3 pigs.

## 4. Discussion

A simple method for measurement of glomerular filtration rate (GFR) in healthy and uremic goats and pigs was developed that is based on iohexol plasma clearance after a single bolus administration. This simplified method was developed by application of a correction factor to a one-compartmental model, based on the terminal elimination phase, to calculate iohexol plasma clearance. The correction factor was derived by comparison of the one-compartment model with the “true clearance” based on a nonlinear mixed effects model.

Our method has several advantages as compared to plasma clearance determined by continuous inulin infusion and measurement of urinary inulin excretion, which currently is the gold standard for GFR measurement [10]. After bolus administration, only three venous blood samples are required at 180, 240 and 300 min without the need for continuous infusion of an exogenous marker, frequent blood sampling during steady-state plasma concentrations or timed urine collections. Moreover, the simplified method, based on sparse sampling, uses standard linear regression, which can be performed in any statistical package, whereas NLME, which is based on more frequent sampling, requires specialized software and personnel. Therefore, this method is easy to perform, less time-consuming and reduces animal discomfort.

To the best of our knowledge, we are the first to develop a method for GFR measurement based on iohexol plasma clearance in goats and pigs. Few studies have measured GFR in pigs using exogenous markers other than inulin [7,21,22,23,24,25] and to our knowledge, there are no such reports in goats. Similar to our study, Luis–Lima has developed a method for calculation of GFR based on blood sampling after bolus injection of iohexol in healthy swine weighing ~150 kg [21]. However, their model has not been validated in animals with impaired kidney function or a body weight similar to humans and requires six blood samples up to 420 min, whereas our method relies on only three blood measurements between 180 and 300 min. One study measured GFR in pigs with impaired kidney function by quantification of urinary iohexol excretion [25]. However, urinary excretion methods are not ideal as urine collection in animals is cumbersome, prone to errors and may require temporary sedation of animals for insertion of an indwelling urinary catheter. In goats, only inulin excretion methods were used [26,27,28,29,30,31,32]. In sheep, which resemble goats, a method for measurement of iohexol plasma clearance was developed, which is based on seven blood samples, whereas our method requires only three blood samples [31].

The Bland–Altman plot showed good agreement between the observed CL (CL_NLME_) and the CL calculated by the simplified method (CL_SM_). The mean ratio was nearly 1, as expected since the simplified method is derived from NLME modeling. However, the simplified method does not take into account interindividual or intra-occasion variability. As such, the standard deviation of the ratio is more important, which was low. This indicates that the simplified method can be used to estimate the true individual clearance in both goats and pigs. Interestingly, when adjusting CL for BSA, a slight bias between the methods occurred, particularly in the higher mGFR range. This may be caused by proportional inaccuracies in the measurement of body weight or the methods for the calculation of BSA.

This study has limitations. First, the relatively limited number of animals may have caused bias by the inclusion of species as a covariate in the model. Second, extrarenal clearance of iohexol was not measured and could theoretically have resulted in an overestimation of GFR. However, Frennby et al. found that extrarenal excretion of iohexol is very limited in anephric pigs (0.087 mL/min/kg) [33]. Therefore, we do not expect that extrarenal clearance has influenced the conclusions of the present study.

## 5. Conclusion

In conclusion, a simple, accurate and minimally invasive method was developed to measure GFR based on plasma clearance of iohexol in healthy and uremic goats and pigs. This method could be used for monitoring GFR in animals during a preclinical drug or medical device development for the treatment of CKD.

## Figures and Tables

**Table 1:** Precision and accuracy of iohexol plasma clearance and mGFR calculated by a nonlinear mixed effects model and the simplified method.

Parameter	Method	Precision (%)	Accuracy (%)	
			P30 *	P10 *
CL	NLME SM	80.9 80.7	53	12
mGFR	NLME SM	49.2 53.6	71	24

CL; iohexol plasma clearance, mGFR, measured glomerular filtration rate, NLME; nonlinear mixed effects model, SM; simplified method. Precision was calculated as: 2 × standard deviation/mean × 100%. * The percentage of SM estimates falling within ±30% (P30) or ±10% (P10) of CL calculated by NLME.

## Data Availability

Data is contained within the article or Supplementary Material.

## Supplementary Materials

The following are available online at https://www.mdpi.com/article/10.3390/biology10060461/s1, Figure S1: Goodness-of-fit plots population PK model, Table S1: Population parameter estimates of the pharmacokinetic iohexol model.
