# Supplementary material for: Simplified Iohexol-Based Method for Measurement of Glomerular Filtration Rate in Goats and Pigs
Source: Biology (Basel). 2021 May 23;10(6):461. doi: 10.3390/biology10060461 (PMC8224736; doi:10.3390/biology10060461)
Supplement: Supplementary file 1 [file biology-10-00461-s001.zip › biology-1230989-supplementary.pdf]

Supplemental Figure

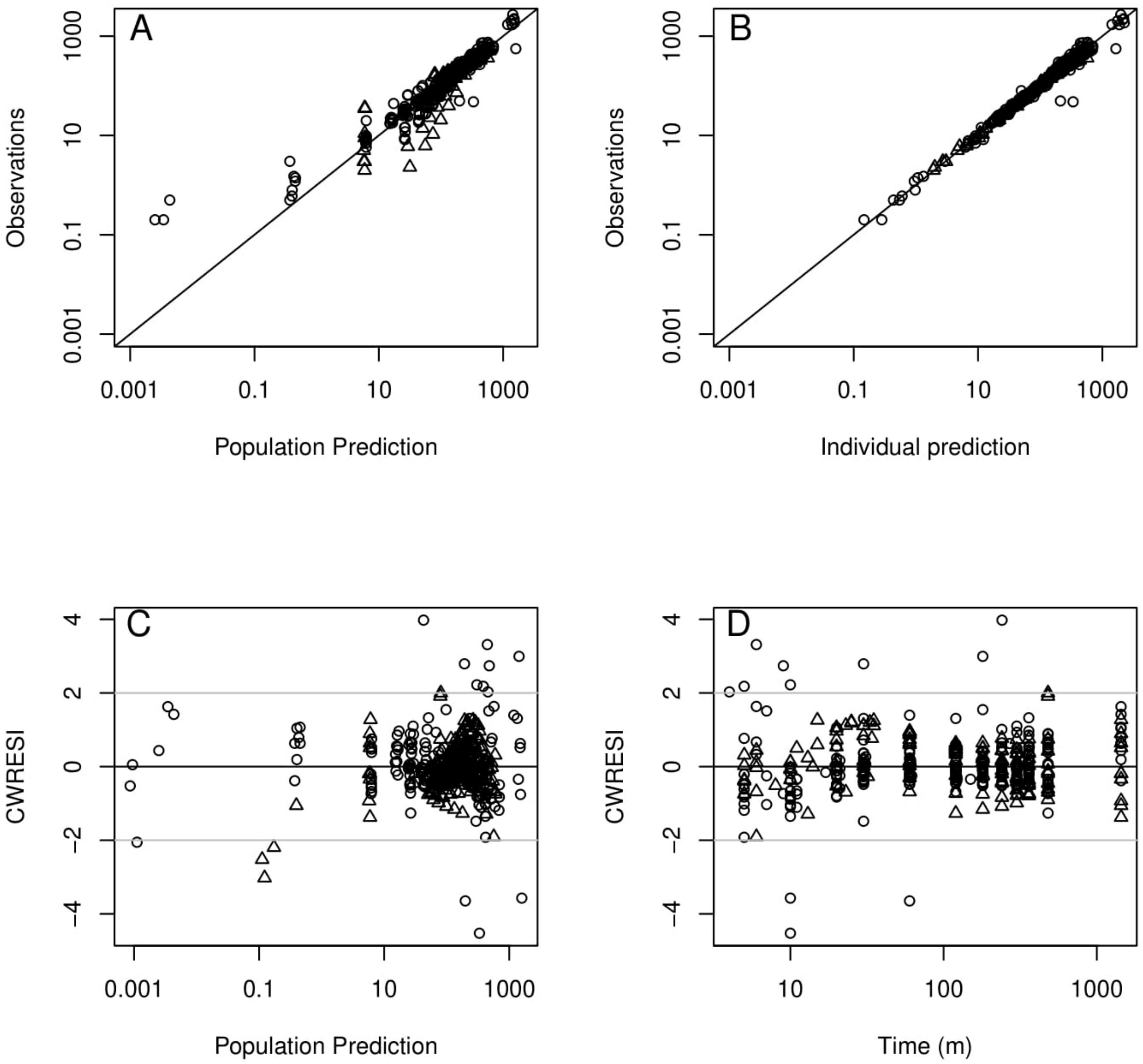

Figure S1. Goodness-of-fit plots population PK model; observed plasma iohexol concentration versus population- (A) and individual (B) prediction (black line = line of unity) and the conditional weighted residuals with interaction (CWRESI) versus population prediction (C) and time (D) (grey line = [-2.2] interval). Goats (triangles), pigs (circles).

Supplemental Table

Table S1. Population parameter estimates of the pharmacokinetic iohexol model

Parameter	Estimate	RSE (%)
$CL_{healthy}$ pig (L/h)	2.92	9.69
V1 (L)	2.57	6.07
Q2 (/h)	0.136	4.56
V2 (L)	0.251	13.5
Q3 (/h)	8.48	9.84
V3 (L)	3.11	8.09
CL_{CKD} pig (L/h)	1.67	10.5
SPEC (goat)	0.568	12.1
	CV (%)	shrinkage (%)
ω^2 IOV CL	32.9	22-44
ω^2 IIV V2	160	22.1
σ^2 prop error	0.036	4.53
σ^2 add error	0.000001*	5.67

*Fixed value, RSE: relative standard error, CV; coefficient of variation, $CL_{healthy}$; iohexol plasma clearance in animals with normal kidney function, CL_{CKD}; iohexol plasma clearance in animals with chronic kidney disease, SPEC; multiplication factor for covariate goat on $CL_{healthy}$ and Cl_{CKD} pig, IOV; interoccasion variability, IIV; interindividual variability, Vx; volume of distribution of compartment x, Qy; intercompartmental clearance of compartment y.
